# Curious Effects of Poisons

**Published:** 1890-12

**Authors:** 


### CURIOUS EFFECTS OF POISONS.

The animal body can be played upon as if it were a machine. The strokes of the central pump, the heart, can be slowed or quickened ; the vital heat lowered or increased ; the pupil of the eye expanded or contracted ; the limbs paralyzed or convulsed ; the blood sent to the surface or withdrawn to the interior ; even the natural hue and color of the body can be changed by the subtle action of various poisons taken into the system.

There is a peculiar offensive sulphur compound called mercaptan. A little of that administered to any one produces the intensest melancholy, tending almost to suicide. We can sometimes detect a similar offensive substance in the breath of patients suffering from melancholia. Similarly, there is a well known poison which produces all the effects of scarlet fever. There is another, a large dose of which brings about all the symptoms of cholera ; and there appear to be several poisons which produce idiocy or actual madness. The Hindoos are said to know a drug which has in Indian history often played the part of a State agent, and has been used to produce imbecility in persons of high rank whose mental integrity was considered dangerous to the despot in power. There is a droll account of the effect produced by a certain root which, in an ancient cloister, the monks once ate by mistake. In the night all were taken with hallucinations, so that the pious convent was like a madhouse. One monk sounded at midnight the matins ; some who, thereupon, thinking it was morn, came into chapel, opened their books, but could not read ; others declaimed ; some sang drunken songs of a character not befitting the place, and the greatest disorder prevailed.

A curious poison is that which Shakespeare makes Friar Laurence give to Juliet as a means of enabling her to escape the proposed marriage with Paris. It would, he assured her, produce temporarily all the symptoms of death—

> Each part deprived of supple government,
> Shall, stiff and stark and cold, appear like death :
> And in this borrowed likeness of shrunk death
> Thou shalt continue two and forty hours
> And then awake as from a pleasant sleep.

Juliet takes the draught, and the effect is precisely as the friar has predicted. It might be supposed that so convenient a poison was purely the invention of a dramatist, and had no sort of equivalent in the drugs of the toxicologist. Modern science, however, has recognized in the contents of the Juliet phial a well known medicine of ancient Greece (*Atropa mandragora*) which really possesses the remarkable power attributed to it in Shakespeare's tragedy. It was actually used by Greek physicians very much as we use chloroform, and under its influence operations were performed. It was known as " death wine," and was in common use till about the fifteenth century, but old medical works are still extant containing descriptions of it, and, a few years ago, this gentleman tells us that a friend of his brought him some of the root from Greece, and by following these old prescriptions he was able to concoct some of this death wine, and to make such experiments with it as to entirely confirm "Friar Laurence's" account of its action. We are further told that when the Jews were under the Romans, and a good many of them were crucified, the Jewish women were in the habit of giving them this mandragora in order to alleviate their sufferings, and it is suggested that as some of the victims were known to have recovered from their apparent death, the practice of breaking the legs was resorted to as an additional safeguard against their restoration.

